# Disabled people faced greater challenges accessing sexual and reproductive health services in the first year of the COVID-19 pandemic in Britain: evidence from the Natsal-COVID survey

**DOI:** 10.1186/s12889-025-24914-3

**Published:** 2025-11-03

**Authors:** Beattie R. H. Sturrock, Emily Dema, Raquel Bosó Pérez, Lorraine Stanley, Jo Gibbs, Pam Sonnenberg, Kirstin R. Mitchell, Catherine H. Mercer, Nigel Field

**Affiliations:** 1 Institute for Global Health, University College London, London, UK; 2 London School of Hygiene and Tropical Medicine, London, UK; 3 MRC/CSO Social and Public Health Sciences Unit, University of Glasgow, Glasgow, UK; 4 Sex With A Difference (SWAD), Dorset, UK

**Keywords:** Disability, Sexual health, Reproductive health, Service access, COVID-19, Britain

## Abstract

**Background:**

Disabled people face barriers to accessing sexual and reproductive health (SRH) services. There is evidence that the general population had difficulty accessing SRH services during COVID-19 but it remains unclear whether disabled people were differentially affected. This study sought to investigate whether people in Britain who reported a disability were more likely to report inability to access SRH services and whether this was associated with functional limitation.

**Methods:**

We analysed data from the National Survey of Sexual Attitudes and Lifestyles (Natsal)-COVID 2 study. This was a cross-sectional, web panel survey of 6,658 18–59-year-old British residents in March–April 2021. Quota-based sampling and weighting were used to achieve quasi-representative population estimates. We defined disability as a long-term physical or mental health condition which affected ones’ ability to carry out day-to-day activities. We calculated adjusted odds ratios (AOR) by reported disability status for wanting but being unable to access ≥ 1 SRH service, wanting but not trying to access SRH services, inability to access different SRH services, and unmet need for condoms. Reasons for, and outcomes after, inability to access services were also investigated.

**Results:**

Participants reporting a disability (unweighted *n* = 1,676), compared to not, were more likely to report wanting but being unable to access ≥ 1 SRH service (AOR 2.23 [1.77–2.82]), inability to access each SRH service type, and an unmet need for condoms. Increasing functional limitation levels were more strongly associated with reporting inability to access ≥ 1 SRH service. Reported disability was associated with higher odds of wanting but not trying to access SRH services (AOR 2.60 [1.50–4.52]) among men but not among women. Participants reporting a disability were more likely to report transport issues and accessing their desired service eventually but not in the way they wanted.

**Conclusions:**

Our study provides evidence that disabled people in Britain were more likely to have difficulty accessing SRH services during COVID-19. However, the extent to which these disparities were created or exacerbated by the pandemic, and whether inequalities persist, is not clear. Our results are relevant for contemporary service design, given that many changes introduced during the pandemic have endured.

**Supplementary Information:**

The online version contains supplementary material available at 10.1186/s12889-025-24914-3.

## Introduction

An estimated 22% of the British population are disabled according to a United Kingdom (UK) Department for Work and Pensions (DWP) survey in 2020–21 [1]. This estimate uses the UK Equality Act 2010 definition of ‘a physical or mental impairment’ which ‘has a substantial and long-term adverse effect on [the] ability to carry out normal day-to-day activities’ [2]. However, disability is complex, heterogenous, and difficult to measure. There may be differences between disabled people^1^ in the nature [3, 4], underlying cause [4], and onset [4] of their disability, affecting their lived experience [3]. Nevertheless, there are important trends in the experience of disability: disability is more common in women [1, 5], people living in deprivation [5–7], non-heterosexual people [8], and older people [5], and these characteristics often intersect to increase marginalisation.

Evidence suggests that disabled people may have poorer sexual health and wellbeing; for example, disabled people are at higher risk of sexual violence [9–14], unsafe sexual behaviours [15] and being diagnosed with a sexually transmitted infection (STI) [14]. Disabled young women were more likely to be worried about their sex lives compared to their non-disabled peers [14] and qualitative evidence suggested that disabled people often feel that they should not express their sexuality [16]. In addition, disabled people experience a variety of barriers to accessing sexual and reproductive health (SRH) services including inaccessible buildings [3], lack of hoists [17], discrimination [3], insufficient staff knowledge [3, 18], and inaccessible sexual health information, for example, using overly complex language [19] or a lack of alternative versions [3]. Moreover, historically, there has been a paucity of data on disability collected by health services [20] making it difficult to understand disabled people’s use of, and access to, services.

During the coronavirus disease 2019 (COVID-19) pandemic, there were widespread changes to SRH service delivery, with increased use of remote services [21, 22]. While this might have increased accessibility, for example, for people who were housebound, it could also have widened gaps, such as for people with hearing impairments [23]. During this period, disabled people reported facing general healthcare barriers including reduced availability of services [24–28], communication issues with telemedicine [25, 27], concerns about privacy during remote consultations [27], transportation problems [27], and being unable to access medications [27, 29, 30]. In addition, there is evidence that the British public found SRH services difficult to access including contraception [31–36], STI testing [36], cervical screening [36, 37] and condoms [34, 36, 38, 39]. The National Survey of Sexual Attitudes and Lifestyles (Natsal) COVID study (a quasi-representative study of the British public with two cross-sectional web-panel survey Waves during COVID-19) has previously shown that, in July–August 2020 (Wave 1), 9.7% of respondents reported trying but being unable to access an SRH service in the four months after the first lockdown in March 2020 [36]. Wave 2 in March–April 2021 also found that the pandemic led to lower-than-expected use of SRH services, such as chlamydia testing and cervical screening, in the year after the first lockdown [40].

Evidence is sparser regarding SRH access for disabled people in Britain during this period. There is some evidence from other countries that disability was associated with worse SRH access during the pandemic [41–43], although the findings are mixed [44, 45]. In Britain, qualitative data suggested that the use of telemedicine in SRH services made access more challenging for people with a hearing impairment or anxiety [23]. In addition, people with anxiety or depression symptoms (who may have identified as disabled) were more likely to report difficulty accessing some SRH services [32, 38]. Information about disabled people’s access to SRH services, and the reasons underpinning this, are important for service planning, improving accessibility, and mitigating inequalities. Given the continuation of many of the SHR service changes made during COVID-19, including remote STI [46] and abortion [47] services, understanding service accessibility during the pandemic is an important gap. The objective of this study was to analyse whether Natsal-COVID wave 2 participants who reported a disability were more likely to report inability to access SRH services and whether this was associated with increasing severity of functional limitation.

## Methods

### Data collection

Natsal-COVID was a study of the SRH of 18–59-year-old British residents during the COVID-19 pandemic through two cross-sectional surveys: Wave 1 (29th July-10th August 2020: investigating experiences in the four months after the first lockdown) [48] and Wave 2 (27th March- 26th April 2021: investigating experiences in the one year after the first lockdown) [49]. Detailed descriptions of the methods for both surveys are available elsewhere [48, 49]. This analysis uses Natsal-COVID Wave 2 data. Data were collected through an online survey run by Ipsos [49]. For the second wave, participants from Wave 1 who had consented to be re-contacted were sampled without quotas [49]. New participants were sampled with quotas for age, gender, region, and social grade. Overall, 6,658 participants took part, of whom 2,098 were re-sampled Wave 1 participants. After weighting for gender, age, region, social grade, ethnicity, and sexual identity, the sample was broadly representative of the general population with some discrepancies [49]. The weighted sample underrepresented people who were married or in good/very good health while the unweighted sample overrepresented people who identified as not heterosexual. This discrepancy was resolved after weighting [49].

### Data analysis

Data analysis was done in STATA 17.0 (College Station, TX: StataCorp LLC.) using complex survey commands to account for weighting. Key variables for participant attributes are defined in Table 1.

**Table 1:** Definitions of participant attribute variables

Term	Definition
Reported disability	Reporting any physical or mental health conditions or illnesses lasting or expected to last for 12 months or more, which reduced their ability to carry out day-to-day activities a little or a lot
Disability limitation	Reporting any physical or mental health conditions or illnesses lasting or expected to last for 12 months or more, which did not reduce their ability to carry out day-to-day activities (long term condition that does not limit), reduced their ability to carry out day-to-day activities a little (disability that limits a little) or a lot (disability that limits a lot)
Sexually experienced	Reporting ever having had oral, anal, or vaginal sex or other genital contact with another person
Sexually active	Reporting sex with another person/people since the start of the first lockdown
Symptoms of anxiety	Scored three or more on the two-item Generalised Anxiety Disorder Scale (GAD-2) [36, 50]
Symptoms of depression	Scored three or more on the two-item Patient Health Questionnaire (PHQ-2) [36, 51]
Gender identity	What the participant felt best described them: male, female, or in another way. If participants selected ‘prefer not to say’, they were routed to the survey exit screen. Natsal-COVID uses inclusive gender identity categories: men including trans men, women including trans women, and people who identify in another way [49]
Sex assigned at birth	Sex the participants reported they were described as at birth: male or female

SRH service access variables (Table 2) were investigated by reported disability status. Participants identifying in another way were included when the denominator included all participants but excluded when adjusting for gender identity in logistic regression due to small numbers (*n* = 20). Reasons for, and outcomes after, inability to access services were investigated by reported disability status. Differences were tested with a chi-squared test.

**Table 2:** SRH service access outcome variables and the denominators used

Service access outcome variables	Description	Denominator used
Wanted but unable to access ≥ 1 SRH service	‘Since the start of the first lockdown, were there any sexual or reproductive health services which you tried to use (for yourself) but couldn’t?’ answered with at least one of: • Contraceptive services/advice • Fertility services/advice • Maternity/antenatal services • Abortion/pregnancy termination services^a^ • Cervical screening (smear test/pap test)^a^ • STI testing • STI follow-up care • HIV testing • Advice or counselling for sexual problems • Relationship support services/advice • Sexual assault/rape support services or helplines • Other sexual or reproductive health services/advice	All participants
Wanted but unable to access a pregnancy-related service	Tried to access at least one of maternity/antenatal or abortion/pregnancy termination services since the start of the first lockdown but couldn’t	Assigned female at birth, sexually experienced and under 45-year-old participants
Wanted but unable to access a reproductive health service	Tried to access at least one of contraceptive or fertility services/advice since the start of the first lockdown but couldn’t	All participants
Wanted but unable to access a sexual or relationship support service	Tried to access at least one of advice or counselling for sexual problems, relationship support services/advice, and/or sexual assault/rape support services or helplines since the start of the first lockdown but couldn’t	All participants
Wanted but unable to access an STI service	Tried to access at least one of STI testing, STI follow-up and/or HIV testing since the start of the first lockdown but couldn’t	Sexually experienced participants
Wanted but unable to access cervical screening	Tried to access cervical screening (smear test/pap test) since the start of the first lockdown but couldn’t	Assigned female at birth participants
Wanted but unable to access other SRH service/advice	Tried to access other type of sexual or reproductive health service/advice since the start of the first lockdown but couldn’t	All participants
Wanted but did not try to access SRH service	Answered with ‘None – I would have liked to, but didn’t try’ when asked if there were any sexual or reproductive health services that they tried to use since the start of the first lockdown but couldn’t	All participants
Unmet need for condoms^b^	Answered yes to ‘Was there any time since the start of the first lockdown when you needed to use condoms, but didn’t because you couldn’t get hold of any because of the pandemic?’	Sexually experienced participants

*SRH* sexual and reproductive health, *STI* sexually transmitted infection, *HIV* human immunodeficiency virus

^a^Question only presented to participants who were trans, assigned female at birth, or did not disclose their sex assigned at birth

^b^Question only presented to sexually experienced participants

We used logistic regression to calculate adjusted odds ratios (AOR), adjusted for age, gender identity, and ethnicity, for wanting but being unable to access to ≥ 1 SRH service and wanting but not trying to access SRH services by reported disability status. Age and gender identity were a priori confounders; adjustment for ethnicity was included after preliminary analysis showed that it resulted in a difference from the crude OR. We also explored several other potential confounders including level of education, relationship status, and social grade – adjusting for these variables did not result in any substantial change to the AOR from the crude OR. We considered whether health status and employment status might confound the association between disability and service access but excluded them as potential confounders given concerns that these were either co-linear with disability or on the causal pathway.

Adjusted Wald tests were used to test significance. We repeated the logistic regression including an interaction term for gender identity to stratify results for men and women. We undertook a sensitivity analysis where participants reporting a long-term condition, but no limitation of day-to-day activities, were excluded (in the main analysis, these participants are included in ‘not reporting a disability’). This compared the odds of inability to access ≥ 1 SRH service in participants with no disability or long-term condition to participants with a limiting disability mirroring analyses as previously done in Natsal-3 [14].

Logistic regression was used to calculate AORs for each SRH service type by disability status. Analyses were adjusted for age, gender identity and ethnicity, except for cervical screening and pregnancy-related services which were adjusted for age and ethnicity and for ‘other SRH services’ which was adjusted for age and gender identity (due to a small number of events restricting the number of parameters). Finally, we calculated AORs for inability to access ≥ 1 SRH service by the level of disability limitation.

## Results

### Participant characteristics

There were 6,658 18–59-year-old participants, and 6,512 (97.8%) had complete disability status and were included in this analysis. Most participants were white and identified as heterosexual or straight, and there was approximate gender balance, reflecting the British population (Additional file 1- Table 3). The weighted prevalence of reported disability was 24.8% (95% CI 23.7–26.0%), corresponding to 1,676 (unweighted) participants, and prevalence was higher in women (26.5% [25.0–28.1%]) compared to men (22.6% [21.0–24.4%]). Disability was, in general, more prevalent in the youngest or oldest age groups, and participants who were white or mixed/multiple/other ethnicity, identified as not heterosexual, were not in employment, or classified their health as bad or very bad, compared to the whole study population. Reporting a disability was equally likely among participants who were sexually experienced compared to not and was more common in those who were not sexually active (prevalence of disability: sexually experienced 25.0% [23.8–26.2%]; not sexually experienced 23.2% [19.4–27.5%]; sexually active 22.2% [20.8–23.6%]; not sexually active 29.6% [27.6–31.8%]. There was no difference in the proportion of participants who had missing data for the main outcome variable (wanted but unable to access ≥ 1 SRH service) by reported disability status (reporting a disability 3.1% [2.2–4.2%], not reporting a disability 2.9% [2.5–3.5%]).

**Fig. 1:**
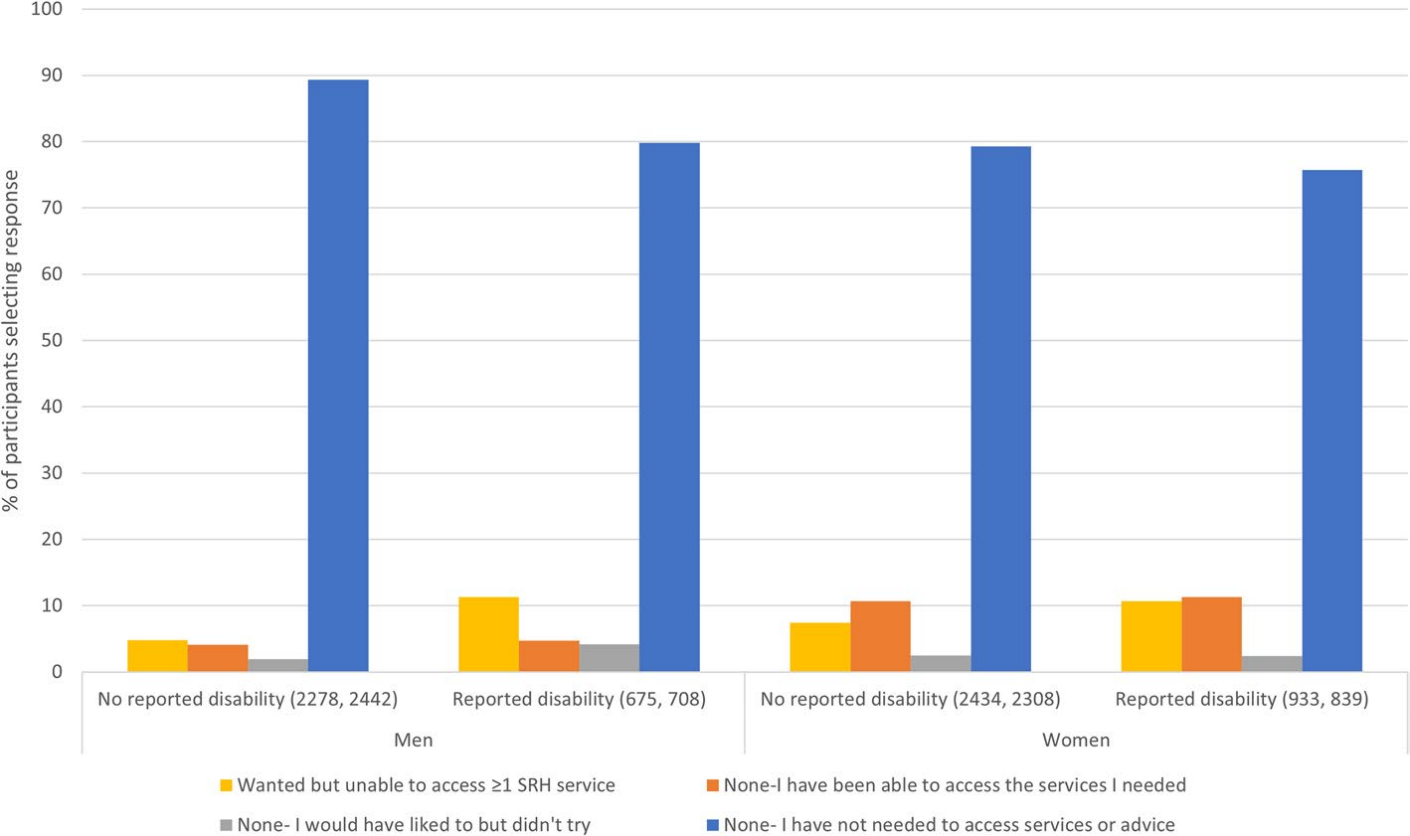
Need for, and access to, SRH services in men and women by disability status. The proportion of 18–59-year-old men and women in Britain reporting different experiences of access to SRH services in the year after the first lockdown by disability status (N unweighted, weighted)

Overall, 7.3% (6.6–8.0%) of the study population reported trying but being unable to access ≥ 1 SRH service in the year after the first lockdown. This varied between men and women and by disability status (Fig. 1). A higher proportion of men and women reporting a disability wanted but were unable to access ≥ 1 SRH service (11.3% [8.8–14.4%] and 10.7% [8.8–12.9%], respectively) compared to those not reporting a disability (4.8% [3.9–5.9%] and 7.4% [6.4–8.6%] respectively). The proportion of participants reporting not needing to access services was similar between groups, although highest in men not reporting a disability.

### SRH service access by disability status

**Fig. 2:**
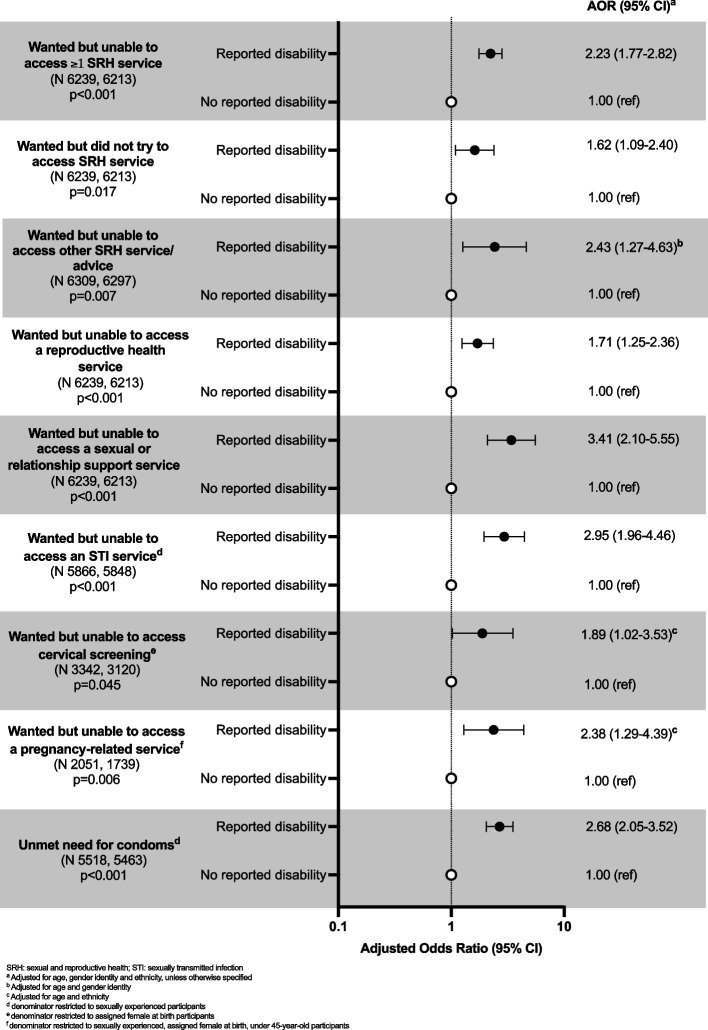
SRH service access by reported disability. Adjusted odds ratios for wanting but being unable to access SRH service and unmet need for condoms in the year after the first lockdown in 18–59-year-olds in Britain by disability status (N unweighted, weighted)

Participants reporting a disability were more likely to report not being able to access ≥ 1 SRH service compared to those without a disability (AOR 2.23 [1.77–2.82]) (Fig. 2). A sensitivity analysis excluding participants who had a non-limiting long-term condition produced similar ORs (Table 3 in Appendix). Reporting a disability was associated with increased odds of being unable to access all SRH service types and having unmet need for condoms. In addition, participants reporting a disability were more likely to report wanting but not trying to access SRH services (AOR 1.62 [1.09–2.40]).

**Fig. 3:**
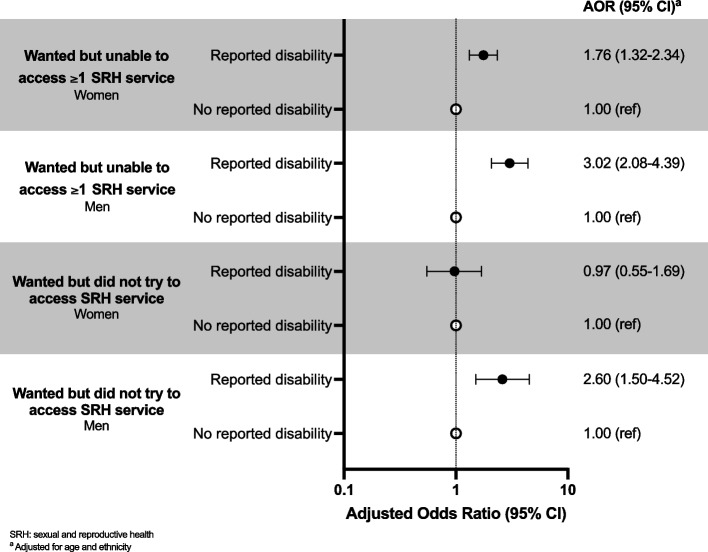
SRH service access in men and women by reported disability. Adjusted odds ratios for wanting but not being able to access ≥ 1 SRH service and wanting but not trying to access SRH services in the year after the first lockdown in 18–59-year-old men and women in Britain by disability status (N unweighted, weighted: 6239, 6213)

Men and women reporting a disability were more likely to report being unable to access ≥ 1 SRH service compared to those not reporting a disability (Fig. 3). The AOR was higher for men (AOR 3.02 [2.08–4.39]) than women (AOR 1.76 [1.32–2.34]). In men, there were higher odds of wanting but not trying to access SRH services in those reporting a disability compared to those without a disability (AOR 2.60 [1.50–4.52]). In women, the odds of wanting but not trying to access an SRH service were similar (AOR 0.97 [0.55–1.69]).

### Reasons for and outcomes after, being unable to access SRH services by disability status

**Fig. 4:**
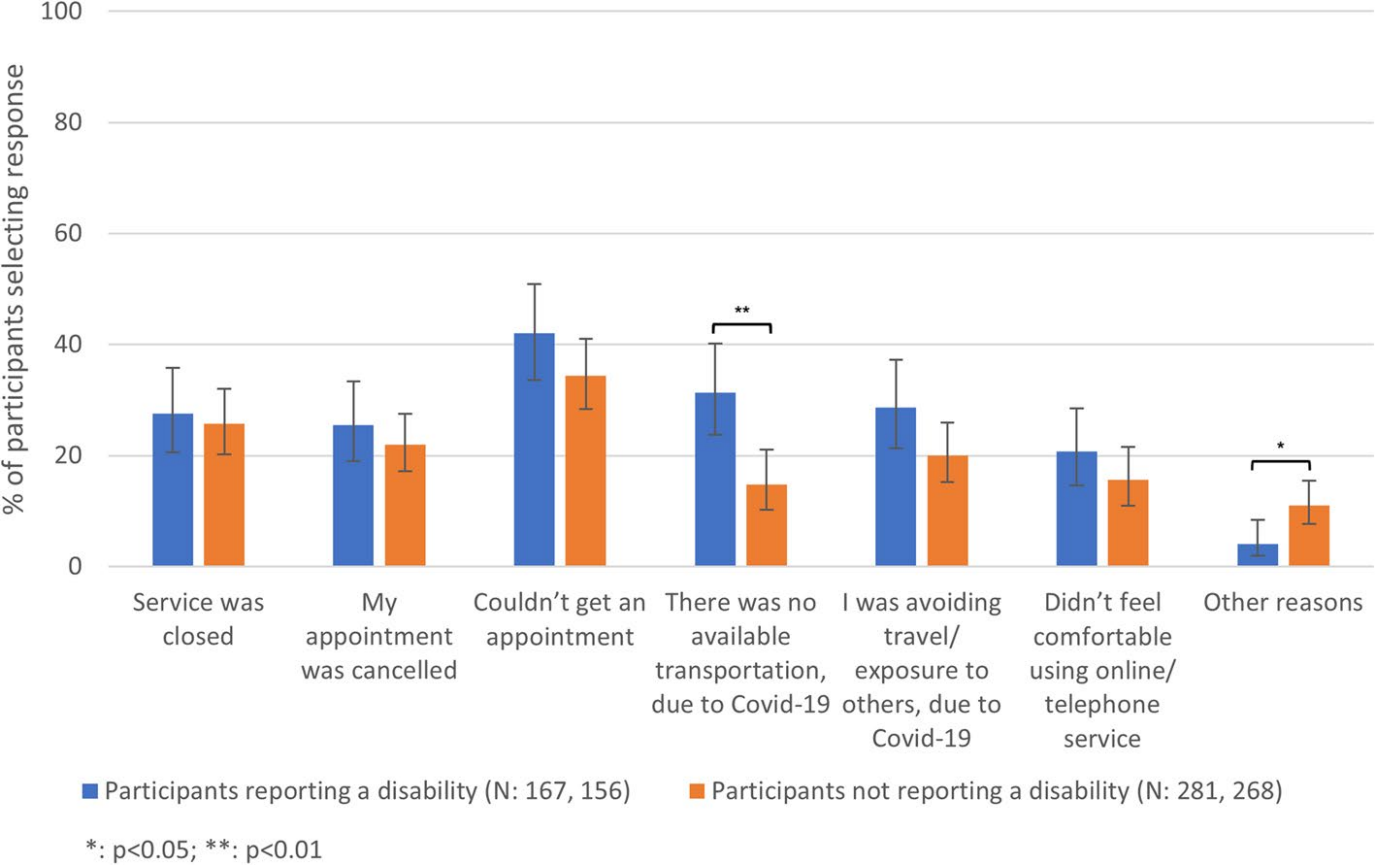
The reasons for being unable to access SRH services by reported disability. The proportion of 18–59-year-olds in Britain reporting different reasons for their inability to access SRH services in the year after the first lockdown by disability status (N unweighted, weighted)

**Fig. 5:**
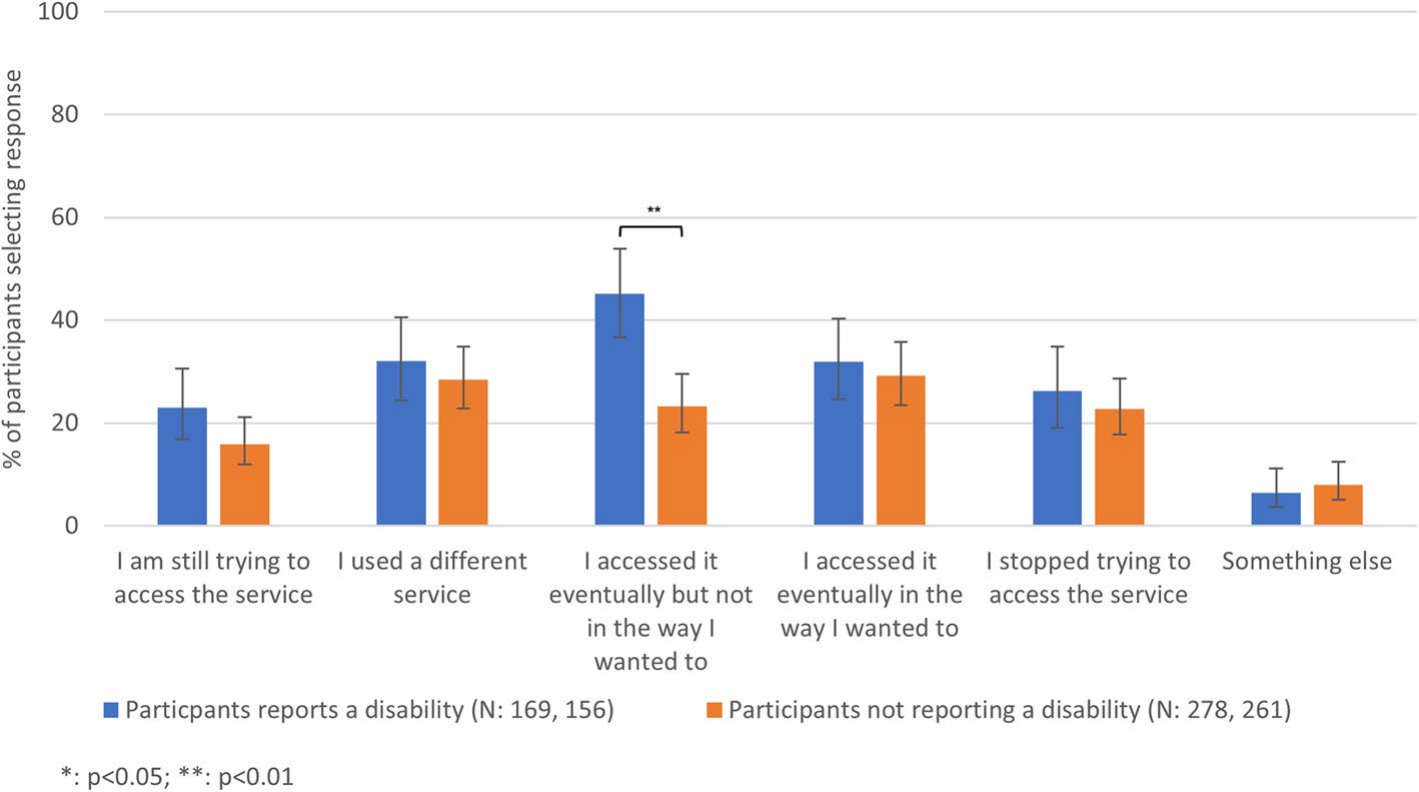
The outcomes after being unable to access SRH services by reported disability. The proportion of 18–59-year-olds in Britain reporting different outcomes after inability to access SRH services in the year after the first lockdown by disability status (N unweighted, weighted)

Compared to those without a disability, participants reporting a disability more frequently reported transport not being available due to COVID-19 (31.3% [23.7–40.1%] vs 14.8% [10.2–21.0%]) and less frequently reported other reasons not specified in the survey (4.1% [2.0–8.4%] vs 11.0% [7.7–15.5%]) (Fig. 4). Participants reporting a disability more frequently reported accessing their desired service eventually but not in the way they wanted to (45.1% [36.6–53.9%] vs 23.3% [18.1–29.5%]) (Fig. 5).

### SRH service access by the level of functional limitation

The level of functional limitation associated with participants’ disabilities was significantly associated with wanting but being unable to access ≥ 1 SRH service (p < 0.001). Compared to those with no disability, participants reporting a disability that limited a little, and a lot, were more likely to report being unable to access ≥ 1 SRH service (AOR 2.00 [1.51–2.65] and AOR 2.76 [1.99–3.85], respectively) while there was no difference for those with a non-limiting long-term condition (AOR 0.95 [0.60–1.49]).

## Discussion

### Findings

Our findings provide evidence of inequities in access to SRH services between disabled and non-disabled people during the COVID-19 pandemic in Britain. Participants reporting a disability were more than twice as likely to report being unable to access ≥ 1 SRH service in the year after the first COVID-19 lockdown and the strength of association increased with increasing functional limitation. This inequity in access was consistent across all types of SRH service and unmet need for condoms.

One explanation for this inequity, suggested by our data, is that accessible transport was not available. Participants reporting a disability were also more likely to report accessing the service eventually but not in the way they wanted to. Potentially, participants reporting a disability may have preferred in-person care given known barriers to telemedicine, such as age [25], cognitive impairment [25], language barriers [25], poor internet connection [27], privacy concerns [27], anxiety [23, 27], hearing impairment [23], and lack of clinician skill [25].

Problems with transportation and telemedicine are just two possible explanations for the association found between disability and inability to access SRH services, and the pathways are likely multifactorial. For example, the public struggled to get consistent information about SRH during the pandemic [23, 34] and such difficulties may be exacerbated for disabled people whose struggles to access SRH information predated COVID-19 [3, 52, 53]. Factors such as employment status, education level, and general health status might have interacted in complex ways to affect how easily disabled people could access services and whether they tried to access required services at all, but our analyses did not have sufficient statistical power to address this.

Our findings are in keeping with existing literature. Various studies have found that disabled people encountered healthcare access barriers during COVID-19 [24–30]. There are also studies from other countries which found that disability was associated with worse SRH care access during COVID-19 [41–43]. Meanwhile, other studies from the United States (US) [44] and Nigeria [45] did not suggest a significant association between problems accessing with SRH care and disability. However, the study populations (postpartum people [44] and women and girls living with HIV [45]) differed to our general population study and there may also have been differences in social attitudes towards disability between Britain, the US, and Nigeria [54], and how COVID-19 lockdowns and service changes were implemented. Previous literature has also suggested that transport was a barrier for disabled people during COVID-19, for accessing healthcare [27] and in general [55]. In addition, our finding of increasing inability to access services with increasing levels of functional limitation is consistent with a study in the US which found that increasing severity of disability was associated with higher odds of foregone medical care compared to no disability, although this study was looking at general healthcare, rather than SRH services [56]. There is also evidence from before the pandemic that disabled people with higher levels of impairment were more likely to be unable to access care [57–59].

### Strengths and limitations

This work addresses a topic for which there is little evidence, and which is important due to the need to reduce health inequalities among an already disadvantaged group. A key strength of this study is the large sample size, which reduces the risk of missing important associations. It was also a general population sample and was quasi-representative [49] making the results more likely to be generalisable. Reassuringly, the prevalence of disability (24.8%) in this analysis was similar to that of the general population (22% [1]). In addition, this study not only explores inability to access SRH care by disability status but also the reasons for, and the outcomes after, this.

However, this was a cross-sectional study, so it is not possible to determine causality, and was not a probability sample. However, despite the drawbacks of a non-probability sample, these are likely to be among the highest quality observational data available on disability and SRH service access during the pandemic. However, all data, including related to disability status and service access, were self-reported with no means of objective validation. We also note that there might be selection bias, such that those who experienced accessibility barriers might be more or less likely to participate in the survey. Other limitations include the risk of digital exclusion [60] and social desirability bias [60] as well as exclusion of people with severe learning difficulties and in institutional settings, thus those with the most severe disabilities [61]. Such exclusions limit the generalisability of our findings to the wider disabled population.

A further limitation is that we were unable to compare SRH service access experience pre-, mid-, and post-pandemic and therefore it is not possible to understand to what extent the access barriers were created by the pandemic (compared to those that existed pre-pandemic) and whether barriers have resolved since the return of services to business-as-usual. Given the time elapsed since the pandemic, it is also more difficult to give contemporaneous recommendations for services based on our study’s findings given that they may not represent the current accessibility of services.

We found that a higher proportion of participants who had used an SRH service in the past year reported a disability compared to the whole population (31.1% compared to 24.8%). However, we were unable to investigate whether the participants reporting difficulty accessing services were the same participants reporting successful use of the same service. Therefore, some reported incidences of successful use may have followed or been alongside unsuccessful use of the same or different services [36]. The proportions of participants reporting ‘none- I have not needed to access any services’ was similar across the analysis population and highest in non-disabled men, in whom reporting not needing to access services might not necessarily represent a lack of need [62]. We hypothesised that the need for SRH services was likely to be approximately the same between comparison groups, although differences in health-seeking behaviour may have affected service use.

There are also challenges in defining disability and some of the participants defined as disabled in this analysis might not have identified themselves as such. A further significant limitation is that our definition of disability is very broad and encompasses physical and mental health conditions, which might affect access to services in different ways. The definition may also exclude people with sensory impairments and learning difficulties, as they may not consider “physical or mental health condition” to be relevant to their experiences of disability. The heterogeneity of disability makes our use of a single measure limited and hampers our ability to interpret the results and provide specific relevant recommendations. Different disabilities will result in different access barriers and thus require different recommendations for mitigations. There is also a breadth of disability severity, which is not adequately captured in the analysis of day-to-day limitation.

## Conclusion

To our knowledge, this is the first study of the British population to show that disabled people had more difficulty accessing SRH care during COVID-19 than those without a disability. Given the continuation of many of the COVID-19 service changes, these findings remain highly relevant. However, these results alone are not sufficient. For example, this analysis does not tell us which of the highlighted disparities were created or exacerbated by the pandemic, to what extent they continue to exist, and how they vary for different types of disability. It was not possible to explore these unanswered questions, given the limitations of the questions asked in the survey and lack of baseline (pre-pandemic) data. Further information on where service access pathways fail disabled people is necessary to inform and implement changes. For this reason, qualitative methods would be valuable to further understand disabled people’s experiences of accessing SRH care. Such qualitative research might consider how different disabilities affect access needs and how these could be mitigated. Longitudinal studies would also be of benefit to investigate the temporal association between different forms of disability and SRH access and outcomes. Such access barriers are unlikely to exist in isolation for SRH services, thus disability could be considered in further studies of general healthcare accessibility, as well as in studies of sexual health.

There is also a role for local SRH services to evaluate their pathways (especially regarding transport options and the use of telehealth) to ensure accessibility. A simple and effective way of improving accessibility would be to routinely ask about accessibility needs at the point of appointment booking. This might help to meet the specific needs of each patient, and improve disability data collection in National Health Service (NHS) services, allowing for more effective monitoring and auditing of accessibility. Services could benefit from an increased focus on accessibility during inspections of health and care facilities [63, 65], incentivising improvement and innovation. Recent recommendations made by Jo’s Cancer Trust, Sex With A Difference, and Healthwatch to improve accessibility of cervical screening are likely to be applicable across SRH services and include ensuring the needs of disabled people are covered in staff training [63]. There might also be benefit from general disability awareness and unconscious bias training for NHS staff to help recognise their own subconscious judgements and how these impact on interactions with patients.

Other recommendations such as creating pathways for people who are housebound [63] and having hoists to facilitate patient transfer [17, 64] are likely to be appropriate, but we are unable to provide data on specific types of disability in our study. Importantly, not all the necessary changes can be made by SRH services alone; a holistic approach is required across healthcare and wider society to ensure public services are accessible to all.

## Supplementary Information

Supplementary Material 1.

## Data Availability

The datasets analysed during the current study are available in the UK Data Archive (serial number SN 8865) [https://beta.ukdataservice.ac.uk/datacatalogue/studies/study?id=8865].

## Appendix

**Table 3:** A comparison between the results of the main analysis and the sensitivity analysis: unadjusted, age-adjusted and fully adjusted odds ratios for wanting but being unable to access ≥ 1 SRH service by reported disability status (main analysis) and reported limiting disability status (sensitivity analysis)

Analysis (N unweighted, weighted)	Reported disability status	OR for wanted but unable to access ≥ 1 SRH service (95% CI)		
		Unadjusted	Age-adjusted OR	AOR (adjusted for age, gender identity, ethnicity)
Main analysis (6239, 6213)	No reported disability	1	1	1
	Reported disability	1.93 (1.55–2.40)	2.17 (1.73–2.73)	2.23 (1.77–2.82)
	*P* value (adjusted Wald test)	< 0.001	< 0.001	< 0.001
Sensitivity analysis (5689, 5693)	No reported disability	1	1	1
	Reported limiting disability	1.88 (1.50–2.35)	2.16 (1.71–2.73)	2.23 (1.75–2.83)
	*P* value (adjusted Wald test)	< 0.001	< 0.001	< 0.001

*SRH* Sexual and reproductive health

## Abbreviations

AOR: Adjusted odds ratio
COVID-19: Coronavirus disease 2019
HIV: Human immunodeficiency virus
Natsal: National Survey of Sexual Attitudes and Lifestyles
NHS: National Health Service
OR: Odds ratio
SRH: Sexual and reproductive health
STI: Sexually transmitted infection
UK: United Kingdom

## Footnotes

1 Language used to describe disability varies, for example person-first (‘people with disabilities’) versus identity-first (‘disabled people’) [66]. We use identity-first language here, in line with the social model of disability [67] (which considers that disability results from an impairment interacting with environmental, social, and political barriers, rather than the impairment itself being the disabler [68]). However, we acknowledge that not everyone will necessarily use, or identify with, this.
